# Annotations

**Published:** 1902-10-25

**Authors:** 


### Annotations.

### The Manchester Infirmary.

For upwards of a quarter of a century Manchester has struggled and squabbled respecting the re-building of its ancient and altogether inadequate infirmary. The non-progressive Board might well express their inefficiency in Scripture language: "We have left undone those things which we ought to have done, and we have done those things we ought not to have done, and there is no health in us." This helpless ineptitude has sadly crippled clinical teaching, and has wrought incalculable suffering on the sick poor. The trustees and leading citizens have at last risen in revolt, and most of the members of the old Board have resigned. On October 16th the following resolution was adopted at a special meeting of the trustees of the Manchester Royal Infirmary, presided over by the Earl of Derby: "That the trustees confirm the policy that there shall be no rebuilding of the infirmary on the Piccadilly site; that that building and site be disposed of on terms to be approved by the trustees and sanctioned by the Charity Commissioners; and that a new infirmary, on plans to be approved by the trustees be built at Stanley Grove without delay, unless a better site can be obtained." We trust that this will pave the way to immediate progress and that Manchester will speedily take steps to secure a great general hospital worthy of the great Lancashire metropolis.

### Draughts v. Fresh Air.

An interesting discussion is in progress in the daily press on the abuse of fresh air. Evidence has been forthcoming which sought to show that the draughty condition of many hospitals was answerable for much discomfort and in not a few instances initiated serious morbid conditions. The long-established fear of fresh air, especially at night is not easily overcome. Observation of houses in our city streets or cottages in the country clearly demonstrates that only comparatively few make any attempt to secure regular admittance of pure air to living and bed-rooms. We are far from approving of "draughts," and we would not deny that such in predisposed people may be capable of producing derangements of physiological processes. But experience and experiment go far in showing that our old ideas regarding the ætiological connection of "colds," pneumonia, pleurisy and the like were based on misconceptions or founded on an incomplete perception of facts. The evidence which comes from sanatoria devoted to the treatment of consumptives amply proves that much of our ancient prejudice regarding the pernicious influence of fresh air needs to be set aside. In the recent discussion to which we have alluded confusion has existed as to the nature of "draughts," and the blame falling on such "aids to discomfort" has sometimes been impartially bestowed on "fresh air" methods also. In these grey days of approaching winter it will be well that in securing protection from "draughts" we also secure free admittance of fresh air, which after all is an essential for securing those conditions of health which may assure the maintenance of a high degree of resistance to "draughts" and all other irritants making for disease.

### The Odours of Disease.

It has long been recognised that both in health and disease characteristic odours occur. The sense of smell has played an important part in the evolution of man. Emanations from animals, exhalations from plants, and even certain substances of an inorganic character possess the property of exciting man's olfactory nerve endings. Physiological and pathological processes are accompanied in many instances by distinctive odours. There are odours peculiar to species, races, families, individuals, and normal states. The growth of many micro-organisms give rise to easily-recognised odours. It must, however, be remembered that the power of detecting odour varies greatly with the animal, the race, the sex, and the individual sensitiveness. Savage races track game by the sense of smell. The Scriptures contain many references to this matter, and clearly indicate that certain races, tribes, and individuals were supposed to possess a characteristic odour. And in the study of disease it is well that the sense of smell should be cultivated. The old physicians were often assisted in their diagnoses by odours; and many an experienced nurse even now will recognise the character of a complaint by the odour emanating from the patient. The clinical study of the subject is one which has been sadly neglected; but it remains rich in possibilities for the careful and judicious clinician. We hope to deal with this matter more fully in a subsequent article. Meanwhile, we shall be glad to hear from any of our readers regarding any exceptional instances in which the sense of smell has proved of service in the diagnosis of disease.

### The Study of Old Age.

Much attention is being devoted to the study of child life. As Professor Sully has so charmingly demonstrated no subject is richer in fascination than the intelligent analysis of children's ways. But in the modern study of evolution much is being gained by adopting the path of retrogression. Instead of worrying with beginnings we may oftentimes track back to an understanding of early states by diligently unravelling the perplexities connected with the latest developments. In medicine there are works innumerable on the affections of childhood, but the books dealing with the conditions of old age are few and altogether inadequate. Charcot, it is true, gave us some valuable studies, and the late Professor Humphry, of Cambridge, furnished a useful analysis, but during recent years few workers have been found in this interesting and but little tilled field of human investigation. Indeed we are in great measure ignorant of the physiology of old age and there are but few clear distinctions between what should be considered as normal retrogression and morbid digression. Even as old age brings about profound changes in physiological processes, so there is reason to believe that senile tissues present certain peculiarities in their reaction to irritants, the exciters of morbid processes. The careful clinician recognises profound differences in the manifestations of disease at the two extremes of life and it would be well if pathologists would endeavour to investigate the conditions underlying such.

